# Couple of the Variational Iteration Method and Fractional-Order Legendre Functions Method for Fractional Differential Equations

**DOI:** 10.1155/2014/928765

**Published:** 2014-01-05

**Authors:** Fukang Yin, Junqiang Song, Hongze Leng, Fengshun Lu

**Affiliations:** ^1^College of Computer, National University of Defense Technology, Changsha 410073, China; ^2^China Aerodynamics Research and Development Center, Mianyang, Sichuan 621000, China

## Abstract

We present a new numerical method to get the approximate solutions of fractional differential equations. A new operational matrix of integration for fractional-order Legendre functions (FLFs) is first derived. Then a modified variational iteration formula which can avoid “noise terms” is constructed. Finally a numerical method based on variational iteration method (VIM) and FLFs is developed for fractional differential equations (FDEs). Block-pulse functions (BPFs) are used to calculate the FLFs coefficient matrices of the nonlinear terms. Five examples are discussed to demonstrate the validity and applicability of the technique.

## 1. Introduction

Fractional calculus plays a significant role in modeling physical and engineering processes which are found to be best described by fractional differential equations (FDEs). Considerable attention has been paid to developing an efficient and fast convergent method for FDEs. Recently, some analytical or numerical methods are introduced to find the solutions of nonlinear PDEs, such as Adomian's decomposition method (ADM) [1, 2], homotopy perturbation method (HPM) [3–5], variational iteration method (VIM) [6–8], orthogonal polynomials method [9–11], and wavelets method [12–17].

Using the operational matrices of an orthogonal function to perform integration and derivative for solving FDEs has received increasing attention. The operational matrix of fractional derivative has been determined for some types of orthogonal polynomials, such as Chebyshev polynomials [18] and Legendre polynomials [9]. The operational matrix of fractional integration has also been determined for Laguerre series [19], Chebyshev polynomials [20], and Legendre polynomials [21]. Recently, Kazem et al. [22] presented the fractional-order Legendre functions (FLFs) and constructed their operational matrix of fractional-order derivatives for the solution of FDEs. The key idea of this technique is that it reduces these problems to those of solving a system of algebraic equations, thus greatly simplifies the problem and can save more computation time. Moreover, the method based on operational matrices of an orthogonal function for solving FDEs is computer oriented.

The variational iteration method (VIM) was first proposed by He [6–8] and has been shown to be a very effective tool for FDEs [23–25]. In order to improve the accuracy and efficiency of the VIM for factional calculus, a modification called fractional variational iteration method (FVIM) [26, 27] was proposed and some successes [28, 29] have been achieved. Moreover, Wu and Baleanu [30], Wu [31] suggested two accurate ways to identify the Lagrange multipliers and various novel variational iteration formulae were obtained for the fractional differential equations. In addition, by using fractional-order Laplace's transform, Yin et al. [32] get a general iteration formula of VIM for fractional heat- and wave-like equations.

Couple of analytical and numerical methods or accuracy and approximation ones is a new trend of developing efficient and fast convergent methods. Recently, Yin et al. [33] developed a modified VIM coupled with the Legendre wavelets, which can be used for the efficient numerical solution of nonlinear partial differential equations (PDEs). Motivated and inspired by the ongoing research in these areas, we extend the above method to FDEs by employing fractional-order Legendre functions, instead of Legendre wavelets. To the best of the authors' knowledge, such approach has not been employed for solving fractional differential equations.

The rest of the paper is organized as follows. In Section 2, we introduce some mathematical preliminaries of the fractional calculus theory and some relevant properties of the fractional-order Legendre functions. In Section 3, the fractional-order Legendre polynomials and their properties are described and nonlinear term approximation by using BPFs is introduced.  Section 4 is devoted to developing MVIM using FLFs. Some numerical experiments are presented in Section 5. Finally, we conclude the paper with some remarks.

## 2. Preliminaries and Notations

Three definitions and one lemma of the fractional calculus theory [35, 36] are listed as follows.

**Definition 1**

A real function *h*(*t*), *t* > 0, is said to be in the space *C*_*μ*_, *μ* ∈ *R*, if there exists a real number *p* > *μ*, such that *h*(*t*) = *t* ^*p*^ *h*_1_(*t*), where *h*_1_(*t*) ∈ *C*(0, *∞*), and it is said to be in the space *C*_*μ*_^*n*^ if and only if *h* ^(*n*)^ ∈ *C*_*μ*_, *n* ∈ *N*.

**Definition 2**

Riemann-Liouville fractional integral operator (*J* ^*α*^) of order *α* ≥ 0 and of a function *f* ∈ *C*_*μ*_, *μ* ≥ −1, is defined as

(1) $$\begin{array}{c} J^{\alpha }f\left(t\right)=\frac{1}{\Gamma \left(\alpha \right)}\int_{0}^{t}{\left(t-\tau \right)}^{\alpha -1}f\left(\tau \right)d\tau ,\;t>0,\\ J^{0}f\left(t\right)=f\left(t\right); \end{array}$$

Γ(*α*) is the well-known Gamma function. Some properties of the operator *J* ^*α*^ can be found, for example, in [35, 36]. One lists only the following, for *f* ∈ *C*_*μ*_, *μ* ≥ −1, *α*, *β* ≥ 0, and *γ* > −1; one has

(2) JαJβf(t)=Jα+βf(t),JαJβf(t)=JβJαf(t),Jαtγ=Γ(γ+1)Γ(α+γ+1)tα+γ.

Because of some defects of Riemann-Liouville derivative in describing real-world phenomena, we will introduce a modified fractional differential operator *D*_*x*_^*α*^ proposed by Caputo [37].

**Definition 3**

The fractional derivative of *f*(*x*) in the Caputo sense is defined as

(3) (Dxαf)(x)={1Γ(m−α)∫0xf(m)(ξ)(x−ξ)α−m+1dξ,(α>0,m−1<α<m),∂mf(x)∂xm,α=m,

where *f* : *R* → *R*, *x* → *f*(*x*) denotes a continuous (but not necessarily differentiable) function.

Some useful formulas and results of Caputo sense derivative are listed as follows:

(4) $$\begin{array}{c} D_{x}^{\alpha }c=0,\;\alpha >0,\;\;c=\text{constant},\\ D_{x}^{\alpha }\left[cf\left(x\right)\right]=cD_{x}^{\alpha }f\left(x\right),\;\alpha >0,\;\;c=\text{constant},\\ D_{x}^{\alpha }x^{\beta }=\frac{\Gamma \left(1+\beta \right)}{\Gamma \left(1+\beta -\alpha \right)}x^{\beta -\alpha },\;\beta >\alpha >0,\\ D_{x}^{\alpha }\left[f\left(x\right)g\left(x\right)\right]=\left[D_{x}^{\alpha }f\left(x\right)\right]g\left(x\right)+f\left(x\right)\left[D_{x}^{\alpha }g\left(x\right)\right],\\ D_{x}^{\alpha }\left[f\left(x\left(t\right)\right)\right]=f_{x}^{\prime }\left(x\right)x^{\left(\alpha \right)}\left(t\right). \end{array}$$

**Lemma 4**

Let *n* − 1 < *α* ≤ *n*, *n* ∈ *N*, *t* > 0, *h* ∈ *C*_*μ*_^*n*^, *μ* ≥ −1; then

(5) $$\begin{array}{c} \left(J^{\alpha }D^{\alpha }\right)h\left(t\right)=h\left(t\right)-\sum_{k=0}^{n-1}h^{\left(k\right)}\left(0^{+}\right)\frac{t^{k}}{k!}. \end{array}$$

## 3. Fractional-Order Legendre Functions

In this section, we first introduce fractional-order Legendre function defined by Kazem et al. in [22], and then derive a fractional integration operational matrix of FLFs. Finally, we give a nonlinear term approximation method.

### 3.1. Fractional-Order Legendre Polynomials

The FLFs are a particular solution of the normalized eigenfunctions of the singular Sturm-Liouville problem

(6) ((x−x1+α)Li′α(x))′+α2i(i+1)xα−1Liα(x)=0,x∈(0,1).

The fractional-order Legendre polynomials, denoted by *FL*_*i*_^*α*^(*x*), are defined on the interval [0,1] and can be determined with the aid of following recurrence formulae:

(7) FL0α(x)=1,  FL1α(x)=2xα−1,FLi+1α(x)=(2i+1)(2xα−1)i+1FLiα(x)−ii+1FLi−1α(x), i=1,2,…,

and the analytic form of *FL*_*i*_^*α*^(*x*) of degree *i* given by

(8) $$\begin{array}{c} FL_{i}^{\alpha }\left(x\right)=\sum_{s=0}^{i}b_{s,i}x^{s\alpha },\;b_{s,i}=\frac{{\left(-1\right)}^{i+s}\left(i+s\right)!}{\left(i-s\right)!{\left(s!\right)}^{2}}, \end{array}$$

where *FL*_*i*_^*α*^(0) = (−1)^*i*^ and *FL*_*i*_^*α*^(1) = 1. The orthogonality condition is

(9) $$\begin{array}{c} \int_{0}^{1}FL_{n}^{\alpha }\left(x\right)FL_{m}^{\alpha }\left(x\right)\omega \left(x\right)dx=\frac{1}{\left(2n+1\right)\alpha }\delta_{nm}, \end{array}$$

where the weight function *ω*(*x*) = *x* ^*α*−1^.

A function *f*(*x*) defined over the interval (0,1] can be expanded as the following formula:

(10) $$\begin{array}{c} f\left(x\right)=\sum_{i=0}^{+\infty }a_{i}FL_{i}^{\alpha }\left(x\right), \end{array}$$

where the coefficient *a*_*i*_ is given by

(11) $$\begin{array}{c} a_{i}=\alpha \left(2i+1\right)\int_{0}^{1}FL_{i}^{\alpha }\left(x\right)f\left(x\right)\omega \left(x\right)dx,\;i=0,1,2,\ldots . \end{array}$$

If the infinite series in (10) is truncated, then it can be written as

(12) $$\begin{array}{c} f\left(x\right)=\sum_{i=0}^{m-1}a_{i}FL_{i}^{\alpha }\left(x\right)=C^{T}\Psi \left(x^{\alpha }\right), \end{array}$$

where *C* and Ψ(*x*) are *m* × 1 matrices defined as

(13) $$\begin{array}{c} C={\left[a_{0},a_{1},\ldots ,a_{m-1}\right]}^{T}, \end{array}$$

(14) $$\begin{array}{c} \Psi \left(x^{\alpha }\right)={\left[FL_{0}^{\alpha }\left(x\right),FL_{1}^{\alpha }\left(x\right),\ldots ,FL_{m-1}^{\alpha }\left(x\right)\right]}^{T}. \end{array}$$

The convergence of fractional-order Legendre polynomials expansion has been discussed in [22].

### 3.2. Integration Operational Matrix of FLFs

The main objective of this section is to generalize the operational matrix of integration for FLFs.

**Lemma 5**

The FLFs Riemann-Liouville fractional integration of *γ* > 0 can be obtained in the form of

(15) $$\begin{array}{c} J^{\gamma }FL_{i}^{\alpha }\left(x\right)=\sum_{s=0}^{i}\frac{b_{s,i}\Gamma \left(1+s\alpha \right)}{\Gamma \left(1+s\alpha +\gamma \right)}x^{s\alpha +\gamma }. \end{array}$$

**Proof**

Consider

(16) JγFLiα(x)=Jγ{∑s=0ibs,ixsα}=∑s=0ibs,iJγ(xsα)=∑s=0ibs,iΓ(1+sα)Γ(1+sα+γ)xsα+γ.

**Lemma 6**

Let *r* > 0; then one has

(17) ∫01JγFLiα(x)FLjα(x)ω(x)dx  =∑s=0 i∑r=0jbs,ibr,j(s+r+1)α+γΓ(1+sα)Γ(1+sα+γ).

**Proof**

Using Lemma 5 and (8), one can have

(18) ∫01JγFLiα(x)FLjα(x)ω(x)dx  =∫01ω(x)FLjα(x)∑s=0ibs,iΓ(1+sα)Γ(1+sα+γ)xsα+γdx  =∫01ω(x)∑s=0 i∑r=0jbs,ibr,jΓ(1+sα)Γ(1+sα+γ)x(s+r)α+γdx  =∑s=0 i∑r=0jbs,ibr,jΓ(1+sα)Γ(1+sα+γ)∫01x(s+r+1)α+γ−1dx  =∑s=0 i∑r=0jbs,ibr,j(s+r+1)α+γΓ(1+sα)Γ(1+sα+γ).

This is proof of Lemma 6.

The Riemann-Liouville fractional integral operator of order *γ* > 0 of the vector Φ(*x* ^*α*^) can be expressed by

(19) $$\begin{array}{c} J^{\gamma }\Phi \left(x^{\alpha }\right)≃P^{\gamma }\Phi \left(x^{\alpha }\right). \end{array}$$

**Theorem 7**

Let Φ(*x* ^*α*^) be FLFs vector; *P* ^*γ*^ is the *m* × *m* operational matrix of Riemann-Liouville fractional integration of order *γ* > 0; then the elements of *P* ^*γ*^ are obtained as

(20) $$\begin{array}{c} P_{i,j}^{\gamma }=\sum_{s=0\;}^{i}\sum_{r=0}^{j}\frac{b_{s,i}b_{r,j}}{\left(s+r+1\right)\alpha +\gamma }\frac{\Gamma \left(1+s\alpha \right)}{\Gamma \left(1+s\alpha +\gamma \right)}\left(2j+1\right)\alpha . \end{array}$$

**Proof**

Using (19) and orthogonality property of FLFs, we have

(21) $$\begin{array}{c} P^{\gamma }=\langle P^{\gamma }\Phi \left(x^{\alpha }\right),\Phi^{T}\left(x^{\alpha }\right)\rangle H^{-1}, \end{array}$$

where 〈*P* ^*γ*^Φ(*x* ^*α*^), Φ^*T*^(*x* ^*α*^)〉 and *H* ^−1^ are two *m* × *m* matrices defined as

(22) 〈PγΦ(xα),ΦT(xα)〉  ={∫01J(γ)FLiα(x)FLjα(x)ω(x)dx}i,j=0m−1  ={∑s=0 i∑r=0jbs,jbr,j(s+r+1)α+γΓ(1+sα)Γ(1+sα+γ)}i,j=0m−1,H−1=diag{(2j+1)α}j=0m−1.

Substituting (22) into (21), one can have (20).

### 3.3. Nonlinear Term Approximation

The FLFs can be expanded into *m*-set of block-pulse functions as

(23) $$\begin{array}{c} \Psi \left(x^{\alpha }\right)=\Phi_{m\times m}B_{m}\left(x^{\alpha }\right). \end{array}$$

Let *t* = *x* ^*α*^ and taking the collocation points as follows:

(24) $$\begin{array}{c} t_{i}=\frac{i-0.5}{m},\;i=1,2,\ldots ,m. \end{array}$$

The *m*-square Legendre matrix Φ_*m*×*m*_ is defined as

(25) $$\begin{array}{c} \Phi_{m\times m}≜\left[\begin{array}{cccc} \Psi \left(t_{1}\right) & \Psi \left(t_{2}\right) & \cdots & \Psi \left(t_{m}\right) \end{array}\right]. \end{array}$$

The operational matrix of product of the Legendre polynomials can be obtained by using the properties of BPFs; let *f*(*t*) and *g*(*t*) be two absolutely integrable functions, which can be expanded in Legendre wavelets as *f*(*t*) = *F* ^*T*^Ψ(*t*) and *g*(*t*) = *G* ^*T*^Ψ(*t*), respectively.

From (23), we have

(26) $$\begin{array}{c} f\left(t\right)=F^{T}\Psi \left(t\right)=F^{T}\Phi_{mm}B\left(t\right),\\ g\left(t\right)=G^{T}\Psi \left(t\right)=G^{T}\Phi_{mm}B\left(t\right). \end{array}$$

By employing Lemma 1 in [38] and (17), we get

(27) f(t)g(t)=(FTΦmm⊗GTΦmm)B(t)=(FTΦmm⊗GTΦmm)inv(Φmm)ΦmmB(t)=(FTΦmm⊗GTΦmm)inv(Φmm)Ψ(t).

## 4. Modified Variation Iteration Method Using FLFs

Consider the following initial value problem:

(28) $$\begin{array}{c} D^{\alpha }u\left(x\right)+N\left[u\left(x\right)\right]+L\left[u\left(x\right)\right]=g\left(x\right),\;\alpha >0, \end{array}$$

(29) $$\begin{array}{c} u^{\left(k\right)}\left(0\right)=c_{k},\;k=0,1,2,\ldots ,m-1,\;\;m-1<\alpha \leq m, \end{array}$$

where *L* is a linear operator, *N* is a nonlinear operator, and *D* ^*α*^ is the Caputo fractional derivative of order *α*.

Wu and Baleanu [30] and Wu [31] applied the VIM to (28), and generalized an accurate variational iteration formula as follows:

(30) un+1=un+∫0xλ(x,τ)(Dαun+N[un]+L[un]−g(x))dτ,                     0<x,  0<α,λ(x,τ)=(−1)α(τ−x)α−1Γ(α),u0=∑i=0m−1uk(i)(0+)xii!+Jα{g(x)}.

Using Lemma 4, (30) can be rewritten as

(31) $$\begin{array}{c} u_{k+1}\left(x\right)=\sum_{i=0}^{m-1}u_{k}^{\left(i\right)}\left(0^{+}\right)\frac{x^{i}}{i!}-J_{\tau }^{\alpha }\left\{Nu_{k}\left(\tau \right)+Lu_{k}\left(\tau \right)-g\left(\tau \right)\right\}. \end{array}$$

However, (31) will generate “noise term” [22, 23] for inhomogeneous equations. Wu [39] gave a new technology to determine the initial iteration value. Motivated and inspired by the work [40–45], we construct an iteration formulae which can accelerate the rapid convergence of series solution when compared with FVIM.

We assume that *g*(*x*) consists of two parts, which can be denoted by *g*(*x*) = *g*_int_(*x*) + *g*_frc_(*x*), where *g*_int_(*x*) is the integer component with respect to *x* and *g*_frc_(*x*) is the fractional component.

The components *u*_0_, *u*_1_,…, *u*_*k*_,… are determined recursively by

(32) u0(x)=∑i=0m−1uk(i)(0+)xii!−Jτα{−gfrc(τ)},u1(x)=u0(x)−Jτα{Nu0(τ)+Lu0(τ)−gint(τ)},⋮uk+1(x)=uk(x)−Jτα×{Nuk(τ)−Nuk−1(τ)+L[uk(τ)−uk−1(τ)]},                       k>1.

In order to improve the performance of FVIM, we introduce FLFs to approximate *u*_*k*_(*x*) and the inhomogeneous term *g*_int_(*x*) as

(33) $$\begin{array}{c} u_{k+1}=C_{k+1}^{T}\Psi \left(x^{\alpha }\right),\;\;g_{\mathrm{int}}\left(x\right)=G^{T}\Psi \left(x^{\alpha }\right), \end{array}$$

in which Ψ(*x* ^*α*^) is defined as (14).

Now for the nonlinear part, by nonlinear term approximation described in Section 3.3, we have

(34) $$\begin{array}{c} Nu_{k}\left(x\right)=N_{k}^{T}\Psi \left(x^{\alpha }\right), \end{array}$$

where *N*_*k*_^*T*^ is matrix of order *m* × 1.

For the linear part, we have

(35) $$\begin{array}{c} Lu_{k}\left(x\right)=L_{k}^{T}\Psi \left(x^{\alpha }\right), \end{array}$$

where *L* is a matrix of order *m* × 1.

Then the iteration formula (32) can be constructed as

(36) $$\begin{array}{c} C_{1}^{T}=-\left(N_{0}^{T}+L_{0}^{T}-G_{\mathrm{int}}^{T}\right)P^{\alpha },\\ C_{k+1}^{T}=-\left(N_{k}^{T}-N_{k-1}^{T}+L_{k}^{T}-L_{k-1}^{T}\right)P^{\alpha }. \end{array}$$

The power of the method depends on the occurrence of the exact solution in the zeroth term. If the exact solution exists in the zeroth component, our method can converge very fast to the exact solution.

## 5. Applications and Results

In this section, we first give two examples to demonstrate that (32) can avoid “noise term” and then use three other examples to illustrate the validity and applicability of our method. The accuracy of our approach is estimated by the following error functions:

(37) $$\begin{array}{c} e_{j}=\text{abs}\left[{\left(u_{\text{exact}}\right)}_{j}-{\left(u_{\text{approx}}\right)}_{j}\right],\\ e=\text{abs}\left(u_{\text{exact}}-u_{\text{approx}}\right). \end{array}$$

**Example 8**

Consider the composite fractional oscillation equation [14]

(38) $$\begin{array}{c} D^{0.25}u\left(x\right)+u\left(x\right)-x^{2}-\frac{2}{\Gamma \left(2.75\right)}x^{1.75}=0,\;0<\alpha <1, \end{array}$$

with the initial condition *u*(0) = 0.

According to (30), the variational iteration formulae can be constructed as

(39) $$\begin{array}{c} u_{n+1}=u_{n}-J^{0.25}\left\{D^{0.25}u_{n}+u_{n}-g\left(x\right)\right\},\\ u_{0}=x^{2}+\frac{\Gamma \left(3\right)}{\Gamma \left(3.25\right)}x^{2.25}. \end{array}$$

The series solution can be obtained as

(40) $$\begin{array}{c} u_{0}=x^{2}+\frac{\Gamma \left(3\right)}{\Gamma \left(3.25\right)}x^{2.25},\;\;u_{1}=x^{2}-\frac{\Gamma \left(3\right)}{\Gamma \left(3.50\right)}x^{2.50},\\ u_{2}=x^{2}+\frac{\Gamma \left(3\right)}{\Gamma \left(3.75\right)}x^{2.75},\ldots . \end{array}$$

One can find that (40) exists “noise term.”

By using the iteration formula (32), we can get the series solution as

(41) u0(x)=x2;u1(x)=u0(x)−Jτα{u0(τ)−x2}=x2;⋮uk+1(x)=uk(x)−Jτα{uk(τ)−uk−1(τ)}=x2,                 k>1;

so we have *u*(*x*) = *x* ^2^, which is the exact solution. Only two iterations are needed to get the exact solution.

**Example 9**

Consider the following initial value problem [46]:

(42) $$\begin{array}{c} D^{0.25}u\left(x\right)+xu^{2}\left(x\right)=\frac{32}{21\Gamma \left(3/4\right)}x^{7/4}+x^{5}, \end{array}$$

with the initial condition *u*(0) = 0.

According to (30), we have the following variational iteration formulae of (42):

(43) $$\begin{array}{c} u_{n+1}=u_{n}-J^{0.25}\left\{D^{0.25}u_{n}+xu_{n}^{2}-g\left(x\right)\right\},\\ u_{0}=x^{2}+\frac{\Gamma \left(6\right)}{\Gamma \left(6.25\right)}x^{5.25}. \end{array}$$

The series solution can be attained as

(44) $$\begin{array}{c} u_{0}=x^{2}+\frac{\Gamma \left(6\right)}{\Gamma \left(6.25\right)}x^{5.25},\;\;u_{1}=x^{2}-\frac{\Gamma \left(3\right)}{\Gamma \left(3.50\right)}x^{2.50},\\ u_{2}=x^{2}+\frac{\Gamma \left(3\right)}{\Gamma \left(3.50\right)}x^{2.50}-\cdots . \end{array}$$

One also can find that (44) exist “noise term”.

By iteration formula (32), we can get the series solution as

(45) u0(x)=x2;u1(x)=u0(x)−Jτα{τu02(τ)−τ5}=x2;⋮uk+1(x)=uk(x)−Jτα{τ[uk2(τ)−uk−12(τ)]}=x2,                  k>1;

so we have *u*(*x*) = *x* ^2^, which is the exact solution. It is easy to deduce that our method is more efficient than the VIM and FIM [46].

**Example 10**

Consider the following fractional Riccati equation [13, 47–49]:

(46) $$\begin{array}{c} D^{\alpha }u\left(x\right)+u\left(x\right)=0,\;0<x<1,\;\;0<\alpha \leq 1,\\ u\left(0\right)=1,\;\;u^{\prime }\left(0\right)=0. \end{array}$$

The exact solution of this problem is *u*(*x*) = ∑_*k*=0_^*∞*^((−*x* ^*α*^)^*k*^/Γ(*αk* + 1)).

**Figure 1:**
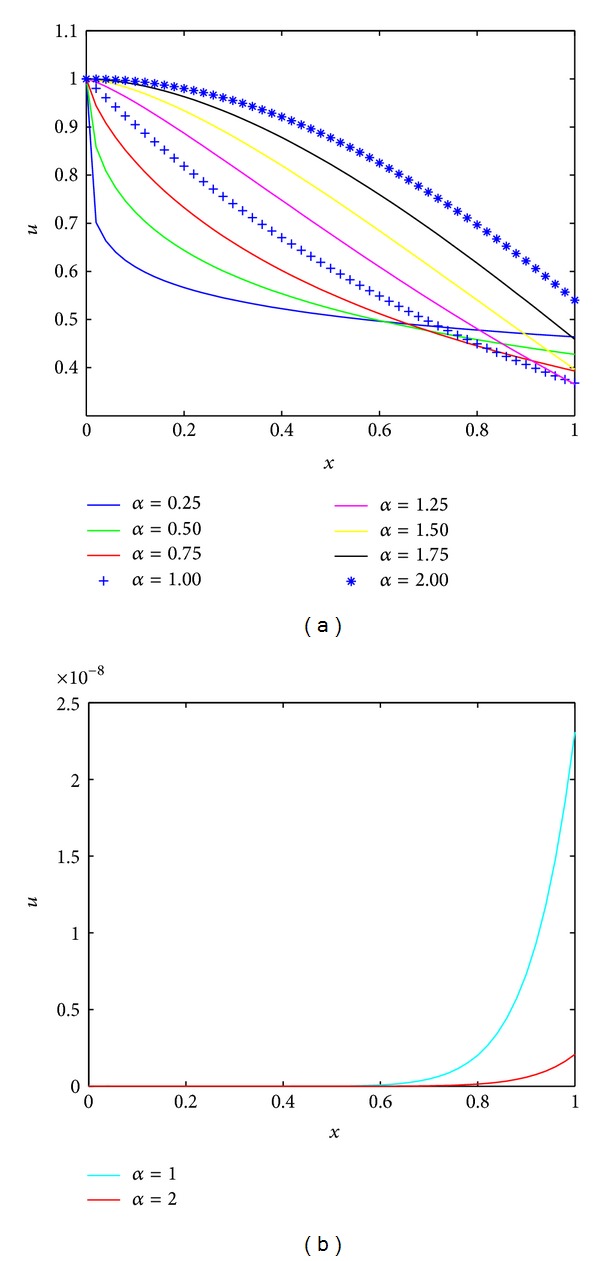
Numerical solutions and error for Example 10.

We solved the problem by applying the technique described in Section 4. When *m* = 9, numerical results are obtained for different values of *α* and shown in Figure 1(a). The absolute errors for *α* = 1.0 and *α* = 2.0 are shown in Figure 1(b). From Figure 1(b), one can see that our method can achieve a good approximation with the exact solution by using a few terms of FLFs.

**Example 11**

Consider the following nonlinear fractional Riccati equation [16, 34]:

(47) $$\begin{array}{c} D^{\alpha }u\left(x\right)+u^{2}\left(x\right)=1,\;0<\alpha \leq 1,\;\;0<x<1, \end{array}$$

subject to the initial state *u*(0) = 0. The exact solution of this problem, when *α* = 1, is *u*(*x*) = (*e* ^2*x*^ − 1)/(*e* ^2*x*^ + 1).

**Figure 2:**
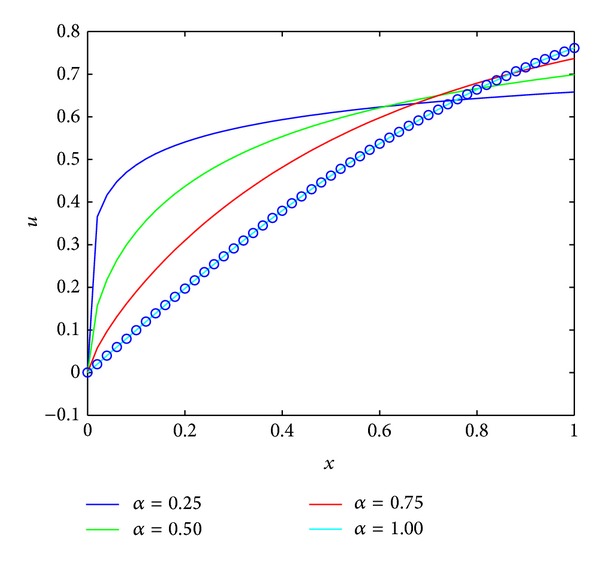
Numerical solutions for Example 11.

We solved the problem, by applying the technique described in Section 4. Numerical results are obtained for different values of *α* when *m* = 9 and shown in Figure 2. In Table 1, we compare our result with [16] for the difference value *α*. From Table 1, one can see that our results are more accurate than those in [16] while only needs a few terms of FLFs.

**Example 12**

Consider fractional Riccati equation [34]

(48) $$\begin{array}{c} D^{\alpha }u\left(x\right)-2u\left(x\right)+u^{2}\left(x\right)-1=0,\;0<\alpha \leq 1 \end{array}$$

subject to the initial state *y*(0) = 0; for *α* = 1 the exact solution is $u\left(x\right)=1+\sqrt{2}\tanh\left[\sqrt{2}x+(1/2)\log\left(\left(\sqrt{2}-1\right)/\left(\sqrt{2}+1\right)\right)\right]$.

**Figure 3:**
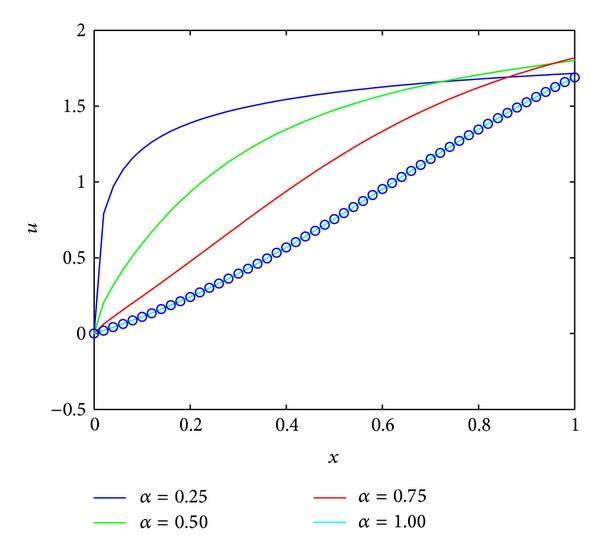
Numerical solutions for Example 12.

We solved the problem by applying the technique described in Section 4.  Figure 3 shows a behavior of the numerical solution for *m* = 9. From Figure 3, it can be seen that our method can achieve a good approximation with the exact solution by using a few terms of FLFs. In Table 2, we compare our result with the modified homotopy perturbation method [34] for the difference value *α*. From Table 2, one can find that our results are more accurate than that obtained by [34]. This demonstrates the importance of presented numerical scheme in solving nonlinear fractional differential equations.

## 6. Conclusion

A new modification of variational iteration method using fractional-order Legendre functions is proposed and successfully applied to find the approximate solution of nonlinear fractional differential equations. The proposed method can avoid the “noise terms” and give approximations of higher accuracy and closed-form solutions if existed. Unlike the VIM, our method can easily overcome the difficulty arising in the evaluation integration and the derivative of nonlinear terms and does not need symbolic computation. In contrast to the FLFs method, our method only needs a few iterations instead of solving a system of nonlinear algebraic equations. Moreover, our method is computer oriented and can use existing fast algorithms to reduce the computation cost.

## Figures and Tables

**Table 1:** Numerical results of Example 11 for *m* = 9 in comparison to [16].

*x*	*α* = 0.50		*α* = 0.75		*α* = 1.00		
	Reference [16]	Ours	Reference [16]	Ours	Reference [16]	Ours	Exact
0.1	0.330159	0.330108	0.190108	0.190101	0.099667	0.099668	0.099668
0.2	0.436737	0.436839	0.309886	0.309976	0.197358	0.197375	0.197375
0.3	0.504842	0.504889	0.404552	0.404615	0.291289	0.291313	0.291313
0.4	0.553802	0.553782	0.481638	0.481632	0.379946	0.379949	0.379949
0.5	0.591265	0.591195	0.545178	0.545090	0.462172	0.462117	0.462117
0.6	0.621026	0.621014	0.597790	0.597783	0.537048	0.537050	0.53705
0.7	0.645480	0.645485	0.641801	0.641820	0.604338	0.604368	0.604368
0.8	0.666016	0.666020	0.678835	0.678850	0.664009	0.664037	0.664037
0.9	0.683560	0.683554	0.710182	0.710175	0.716300	0.716298	0.716298

**Table 2:** Numerical results of Example 12 for *m* = 9 in comparison to [34].

*x*	*α* = 0.50		*α* = 0.75		*α* = 1.00		
	Reference [34]	Ours	Reference [34]	Ours	Reference [34]	Ours	Exact
0.1	0.321730	0.592833	0.216866	0.245446	0.110294	0.110308	0.110295
0.2	0.629666	0.933104	0.428892	0.475051	0.241965	0.241990	0.241977
0.3	0.940941	1.174069	0.654614	0.710050	0.395106	0.395119	0.395105
0.4	1.250737	1.346694	0.891404	0.938523	0.568115	0.567830	0.567812
0.5	1.549439	1.473790	1.132763	1.149016	0.757564	0.756032	0.756014
0.6	1.825456	1.570577	1.370240	1.334339	0.958259	0.953583	0.953566
0.7	2.066523	1.646302	1.594278	1.491949	1.163459	1.152968	1.152949
0.8	2.260633	1.706644	1.794879	1.622950	1.365240	1.346381	1.346364
0.9	2.396839	1.756349	1.962239	1.730575	1.554960	1.526927	1.526911
